# Supplementary figures and images for: Target-selective vertebrate motor axon regeneration depends on interaction with glial cells at a peripheral nerve plexus
Source: PLoS Biol. 2023 Aug 17;21(8):e3002223. doi: 10.1371/journal.pbio.3002223 (PMC10464982; doi:10.1371/journal.pbio.3002223)

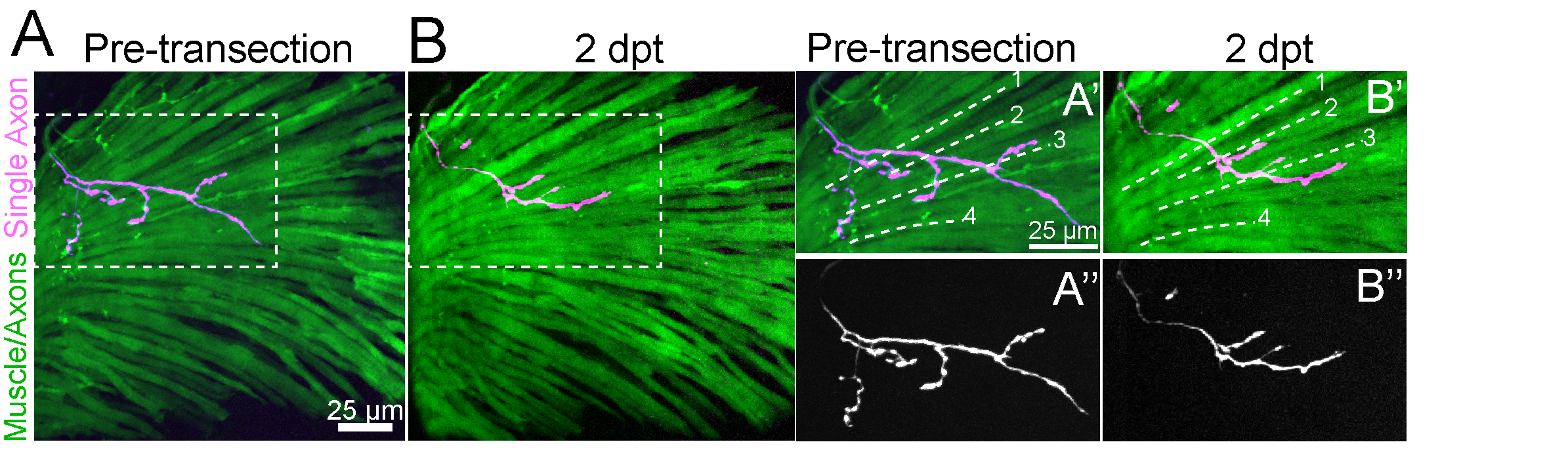

Supplement: S1 Fig — (A) Maximum projection prior to axon transection of abductor muscle in Tg(α-actin:GFP);Tg(mnx1:GFP) larvae showing muscles and motor axons (green motor axons are faint in this example) with a single axon trajectory in magenta. (B) After 2 days, this labeled regenerated axon has reoccupied its original muscle fibers but has formed unique branches. Insets are expanded in A’ and B’ with individual muscle fibers labeled with the dotted lines to compare the axon location. The single labeled axon is shown in A” and B”. (TIF) [file pbio.3002223.s006.tif]

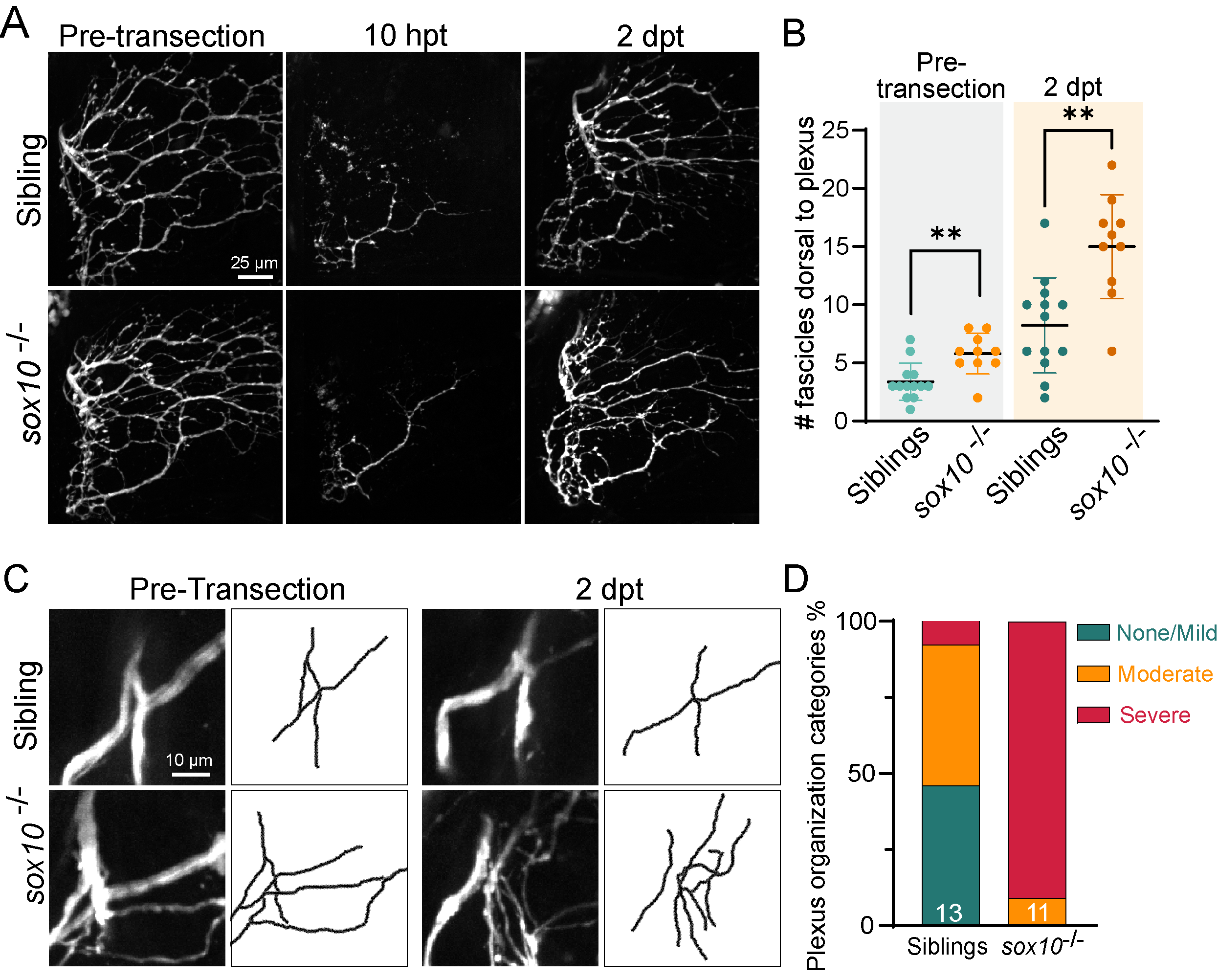

Supplement: S2 Fig — (A) Maximum projections of abductor muscle innervation labeled with Tubb:dsRed in sibling and sox10cls mutant larvae. The same fin is shown pre-transection, at 10 hpt (axon degeneration), and at 2 dpt (axon regeneration). (B) sox10 mutants display a subtle increase in axon defasciculation, as measured by a higher number of individual axon fascicles quantified dorsal to the dorsal plexus, prior to axon transection that increases after axon regeneration. (C) Maximum projection through the plexus region before transection and at 2 dpt. sox10 mutants display a disordered plexus region that worsens after injury. (D) sox10 mutants more frequently display severely disorganized axon patterning at the plexus at 2 dpt; ** p < 0.01. (TIF) [file pbio.3002223.s007.tif]

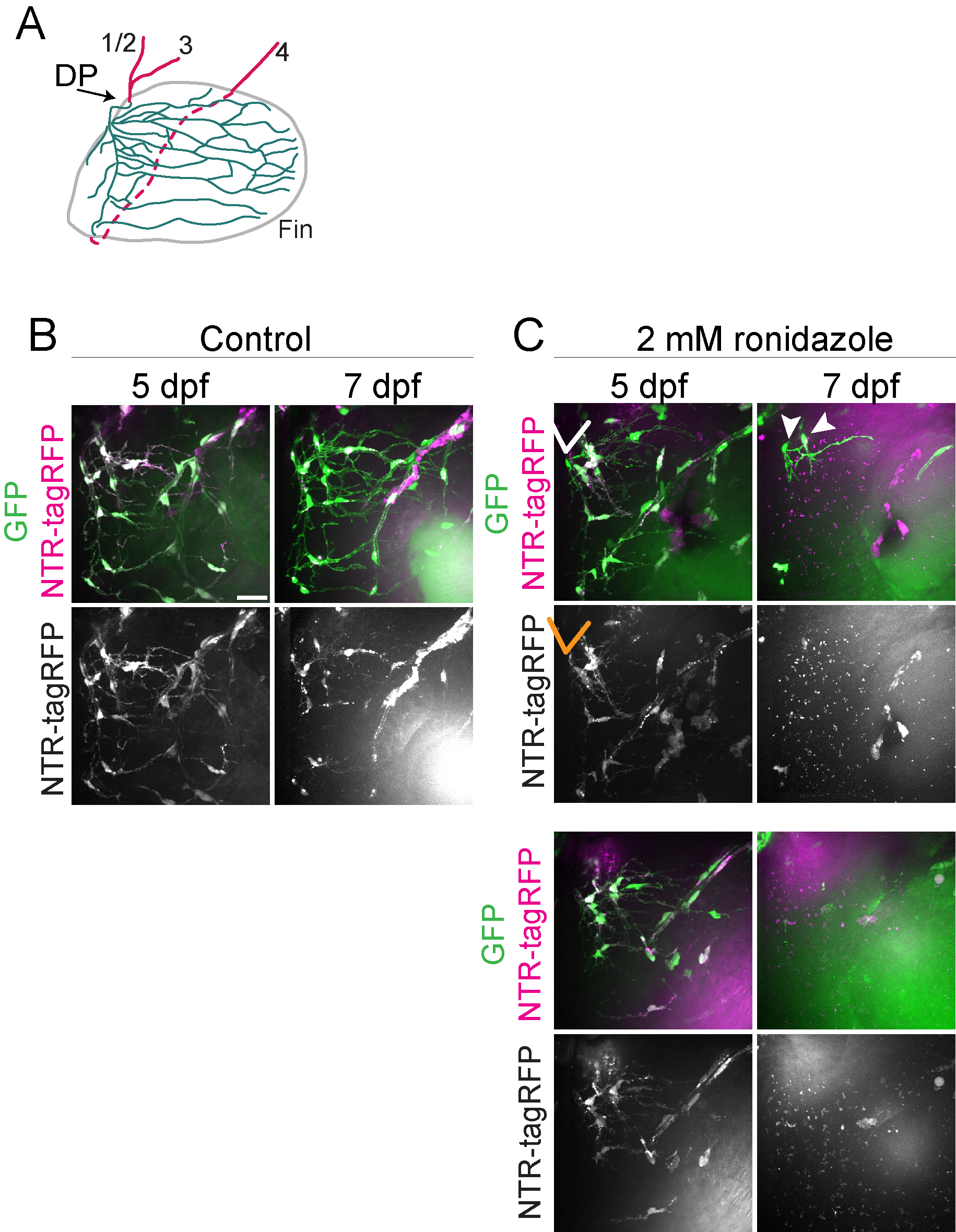

Supplement: S3 Fig — (A) Schematic of innervation showing region included B and C. DP = Dorsal plexus. Nerves 1–4 are labeled. (B) Maximum projection through pectoral fin of control 37a>EGFP, NTR-tagRFP at 5 and 7 days post fertilization (dpf). (C) Maximum projection through 2 examples of 37a>EGFP, NTR-tagRFP larvae treated with ronidazole to ablate Schwann cells. The arrow points to a GFP-labeled Schwann cell that does not express NTR-tagRFP and is spared from ablation at 7 dpf (arrowheads). Schwann cell-ablated animals display tagRFP-positive debris (puncta) at 7 dpf. Scale bars are 25 microns. Original data for panels B, D are in S1 Data. (TIF) [file pbio.3002223.s008.tif]
